# Testing adaptive hypotheses for an evolutionarily conserved trait through slow-motion videos of pollinators

**DOI:** 10.1098/rsos.251127

**Published:** 2025-09-24

**Authors:** Robin Waterman, Sally Song, Nicholas Bhandari, Jeffrey K. Conner

**Affiliations:** ^1^ Kellogg Biological Station, Plant Biology, Michigan State University, East Lansing, MI, USA; ^2^ Biological Sciences, Wellesley College, Wellesley, MA, USA

**Keywords:** tetradynamy, anther separation, stamen differentiation, stamen dimorphism, Brassicaceae, pollination efficiency, wild radish, *Raphanus raphanistrum*, pollinator specialization, heteranthery

## Abstract

Traits conserved across evolutionary time often provide compelling examples of key adaptations for a given taxonomic group. Tetradynamy is the presence of four long stamens plus two short stamens within a flower and is conserved across most of the roughly 4000 species in the mustard family, Brassicaceae. While this differentiation in stamens is hypothesized to play a role in pollination efficiency, very little is known about the potential function of the two stamen types. The present study sheds new light on this mystery using wild radish (*Raphanus raphanistrum*), a widespread and well-studied tetradynamous plant. We used data collected from slow-motion videos of pollinators visiting wild radish flowers to test three adaptive hypotheses (not mutually exclusive): (H1) short and long stamens are specialized for either feeding or pollinating; (H2) short and long stamens are specialized for different pollinator taxa; and (H3) the presence of short and long stamens increases pollinator movement and thus effectiveness. We find evidence consistent with hypothesis H3, but no evidence for hypotheses H1 or H2. Thus, tetradynamy may be an adaptation for generalized pollination, enabling effective visits by the variety of pollinators visiting most species of Brassicaceae.

## 1. Introduction

While biologists often focus on trait divergence, trait similarities are an important feature of taxonomic groups at various levels. When traits diverge slower within than among taxonomic groups, they may be considered evolutionarily conserved. Hypotheses to explain these slowed rates of evolution include a lack of additive genetic variance [1], constraints [2] and selection to maintain current trait means [3].

Flowering plants first evolved approximately 140 million years ago, diversifying into most extant families by around 100 million years ago [4]. In that time, flowers have evolved diverse and complex traits that have long fascinated evolutionary biologists. In plants that rely on animal pollinators to reproduce, the evolution of floral form and function is often shaped by plant–pollinator interactions. Some of these adaptations attract pollinators with visual and/or olfactory cues [5], while others reward pollinators with pollen and nectar [6], and still others increase the efficiency of pollen transfer from anther to stigma by promoting contact with pollinator bodies [7]. Stamens can serve critical functions in all three of these components of pollination.

While some plant species possess uniform stamens within a flower, it is also common for stamens within a flower to be differentiated in one or more traits including length, orientation, colour, sterility and maturation time. When such trait differences accompany functional differences, flowers are often termed ‘heterantherous’ [8]. A well-studied example of heteranthery comes from nectarless, bee-pollinated flowers with a ‘division of labour’ between short conspicuous stamens specialized for feeding and long cryptic stamens specialized for pollen export [9]. Another example comes from stamens that differ in anther dehiscence time in *Clarkia*, *Digitalis* and *Axinaea*, which are thought to function by gradually presenting pollen to pollinators, preventing pollen from being depleted too quickly [10–12].

**Figure 1.**
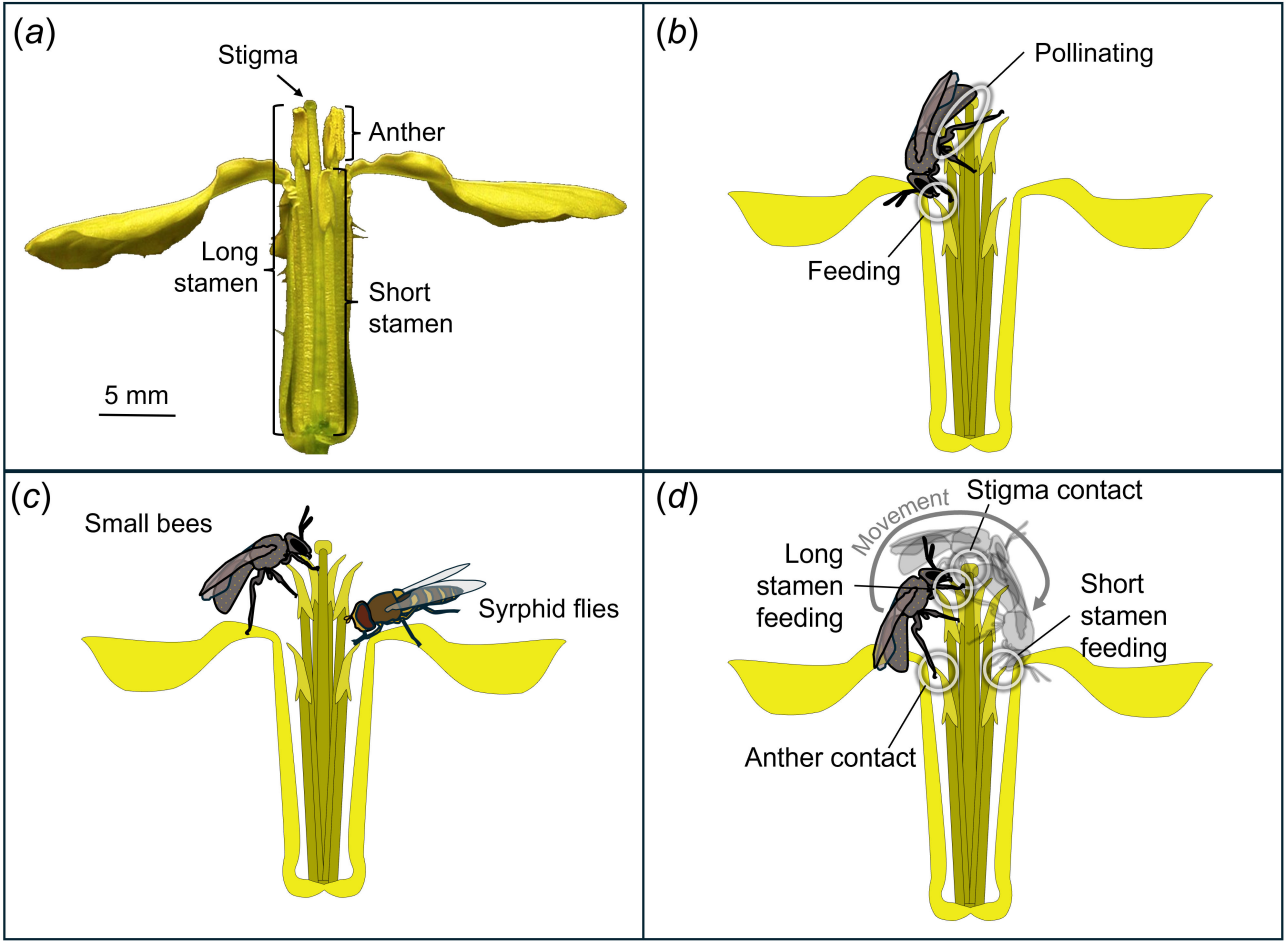
(*a*) Photo of a wild radish flower with one sepal removed to help reveal anatomy; labels point to the pollen-receiving stigma, one of the six pollen-producing anthers, one of the four long stamens and one of the two short stamens. (*b*–*d*) Conceptual diagrams of the three research hypotheses: (*b*) division of labour, (*c*) pollinator specialization and (*d*) movement.

In contrast, little is known about the possible function of intrafloral stamen differentiation in tetradynamous flowers, those with four long and two short stamens (figure 1*a*). Tetradynamy is phylogenetically conserved, occurring across the majority of the nearly 4000 Brassicaceae species (including basal *Aethionema*) and considered diagnostic for the family [13,14]. Tetradynamy likely evolved when Brassicaceae split from its sister clade Cleomaceae [15], which generally has six equal-length stamens [16]. Measures of selection on the difference in length between long and short stamens (‘anther separation’) in wild radish have found evidence for both stabilizing [17] and disruptive selection [18] through male fitness. In both cases, there is selection to maintain current trait means, which lends support to the hypothesis that tetradynamy is maintained by selection. Furthermore, the evolution of tetradynamy does not appear to be constrained, based on a rapid response to artificial selection [19] and reversion of species from at least two Brassicaceae genera to equal-height stamens [14]. Despite this evidence that tetradynamy is conserved across evolutionary time through selection, the functional advantage of having two stamen types of differing lengths within flowers remains unclear.

Prior studies have found that long stamen anthers in *Brassica rapa* flowers produce more pollen per anther than short stamen anthers [20], but the opposite pattern exists in *Raphanus raphanistrum* [17]. In both species, pollinators remove a greater proportion of the pollen produced by long stamen anthers per visit [17,20]. Additionally, the presence of short stamens in experimentally manipulated flowers increased pollinator visit duration in *B. rapa* [20], and flowers with longer short stamens relative to the population mean in *R. raphanistrum* had slightly higher rates of pollinator visitation [21].

Gradual pollen presentation, in which pollen is dispensed over time to avoid depletion [11,22], is unlikely to explain tetradynamy in wild radish because short and long stamens do not differ in pollen dehiscence time (J.K.C. 2014, unpublished data), and measures of selection on anther separation across days differing in pollinator visitation rate did not support this hypothesis [18]. Although neither short nor long stamens produce sterile feeding pollen or are brightly coloured to attract pollinators, it remains possible that a division of labour between feeding and pollinating occurs [9]. Another possibility is that long and short stamens are specialized to make better contact with different pollinator taxa. Pollinators may be acting as multiple selective agents with different adaptive peaks for stamen length [23], allowing a plant with multiple stamen lengths to be simultaneously well adapted to multiple pollinators.

In this study, we used wild radish as a model system to investigate the functional significance of tetradynamy. We built upon our prior studies by employing data from slow-motion videos of pollination events in the field to test two *a priori* hypotheses that are not mutually exclusive: (H1) short and long stamens specialize on either feeding or pollinating (hereafter ‘division of labour’; figure 1*b*); and (H2) short and long stamens specialize on different pollinator taxa (hereafter ‘pollinator specialization’; figure 1*c*). Initial analyses of our data prompted us to formulate and test a third hypothesis: (H3) the presence of short and long stamens increases pollinator movement on the flower and thus effectiveness (hereafter ‘movement’, figure 1*d*). Specifically, we propose that when pollinators move from feeding on one stamen type to another during a visit, their bodies are more likely to contact the anthers and stigma.

## 2. Methods

### 2.1. Study species

Wild radish (*Raphanus raphanistrum* ssp. *raphanistrum*) is a globally distributed agricultural weed native to the Mediterranean region [24] with four long medial stamens and two short lateral stamens (figure 1*a*). Anthers on short and long stamens do not differ in colour, but anthers on short stamens are slightly longer than those on long stamens (short = 2.31 ± 0.08 mm, long = 2.11 ± 0.08 mm). Wild radish is self-incompatible and relies on insect pollinators for reproduction. The species is visited by at least 15 genera of insects from at least three orders (Hymenoptera, Lepidoptera and Diptera) in both Europe and North America [25], with 14 of those genera effectively pollinating, based on seed set [26]. Small bees (primarily *Dialictus*) and syrphid flies (primarily *Toxomerus*) constitute the vast majority of visitors to wild radish in the Midwest [25], including in the present study’s location (79% of 84 visits in preliminary observations by Robin Waterman and Nicholas Bhandari), so we focused our data collection efforts on these two taxa.

### 2.2. Plant material

The wild radish seeds were from the North American weedy ecotype, used in many previous studies of this species (e.g. [27–29]). In order to create an experimental group of plants containing a large range of variation in stamen traits, seeds were sourced from a natural population (‘BINY’ originally collected from Binghamton, New York) and derived populations artificially selected for low anther separation [19] and both low and high anther exsertion [30]. The final sample included 64 individual plants (2021: 27; 2022: 37) from 24 full-sibling families, with 17 BINY plants, 46 separation-selected plants and 1 exsertion-selected plant (due to poor germination in these seeds). Plants were grown to maturity in 6′ pots filled with SureMix (Michigan Grower Products) in a greenhouse at the W. K. Kellogg Biological Station in Hickory Corners, MI (42°24′ N, 85°24′ W, 288 m elevation).

### 2.3. Slow-motion video collection

During each of 17 field observation days across the summer growing season (2021: 10; 2022: 7), flowers from a subset of plants were monitored for visits by naturally occurring small bees or syrphid flies in old field habitats at the Kellogg Biological Station. In 2021, plants were moved outside only while being observed, while in 2022, plants remained outside between the first and last observation days. Flowers were not bagged prior to visits in either year in order to better mimic natural conditions. Wild radish flowers last for 1–2 days and are quickly replaced by more, which allowed for observations of newly opened flowers across multiple days from the same set of plants. Pollination events were opportunistically recorded in slow motion (1080 pixels at 240 frames per second) using either an iPhone XR or iPhone 11 camera with a macro lens attachment (Apexel HD 10× Macro Lens; see electronic supplementary material, video S1 for example). A total of 136 videos were included in the final dataset (2021: 76; 2022: 60), capturing 65 small bee and 71 syrphid fly visits.

In an effort to validate that our video-based anther contact measures translate to physical placement of pollen on pollinators, we conducted a small preliminary test in which we brushed long and short anthers with different colours of fluorescent dye (Day-Glo Eco21 and Radiant Color TI-CH6610) on a subset of flowers. Following Natalis & Wesselingh [31], we then collected three small bees immediately after they visited a marked flower, anaesthetised them on ice, and swabbed them with gelatin cubes, separately for their side, ventral and dorsal body sections. We melted each gelatin cube onto a microscope slide and counted dye particles under a compound light microscope (*n* = 2104 particles total).

### 2.4. Video processing

For each video, the variables recorded were pollinator taxa (small bee or syrphid fly); total duration of contact between three pollinator body sections (see details below) and long and short stamen anthers; presence or absence of contact between pollinator body sections and long stamen anthers, short stamen anthers and stigmas; and feeding behaviour (short stamen pollen, long stamen pollen or nectar). For each video, we also recorded the number of times the pollinator moved from feeding on one stamen to another of the same or different type. These variables were recorded through close observation of video footage by the same experimenter for each variable to avoid bias. Unlike anther contact, stigma contact was usually very brief, so we only recorded presence/absence of contact.

We chose to examine anther contact with different body sections to serve as another indicator of how successfully the flower transfers pollen to pollinators. The three body sections were chosen based on a preliminary analysis of 82 videos for binary anther contact using 11 body sections: legs, wings, ventral head, side head, dorsal head, ventral abdomen, side abdomen, dorsal abdomen, ventral thorax, side thorax and side thorax. There was negligible contact with the dorsal abdomen, dorsal thorax and wings. Among the remaining body sections, hierarchical clustering revealed three groups: legs; ventral head, ventral abdomen and ventral thorax (hereafter ‘ventral’); and side head, dorsal head, side abdomen and side thorax (hereafter ‘side’). Anther contact was not strongly correlated among the resulting clustered sections (|Pearson’s *r*| = 0.05, 0.09 and 0.32).

### 2.5. Statistical analysis

All statistical analyses were done in RStudio running R v. 4.4.2 [32]. The package ‘nnet’ [33] was used to generate multinomial models. The package ‘emmeans’ [34] was used to test significance of model terms (type III analyses of variance/deviance), conduct post hoc analyses on interactions, and generate estimated marginal means. All plots were made using the package ‘ggplot2’ [35].

To test for differences in the proportions of visits by pollen feeding type (only long versus only short versus both long and short stamen anthers), we used pairwise Fisher’s exact tests (‘fisher.test’ function). To test for differences between pollinators (small bees versus syrphid flies) in feeding type, we conducted a multinomial logistic regression (‘multinom’ function) and assessed significance with a likelihood ratio test (‘anova’ function).

To test for differences in anther contact, we quantified contact in three ways: contact or no contact (binary variable), number of body sections contacted (discrete count variable) and duration of contact (continuous variable). We used these as response variables in generalized linear models (‘glm’ function) with binomial, Poisson and normal distributions, respectively. The predictor variables were stamen type (long versus short), pollinator (small bee versus syrphid fly), feeding type and all two- and three-way interactions. For the binary and count models, we had insufficient power to resolve the feeding type by stamen type interaction, so we dropped it and the three-way interaction from these models. Consequently, we tested the *a priori* hypothesis that there would be greater contact with long stamens during short stamen feeding and vice versa using Fisher’s exact tests.

Just 4 of 136 visits were nectar foraging only, and an additional 5 were nectar and pollen foraging, so we did not include foraging mode in the analyses. Excluding these nine points from the analyses did not qualitatively change the results. For duration, we also ran a separate model in which we added body section (legs versus ventral versus side) and all two-, three- and four-way interactions with it as predictors.

We ran similar models for stigma contact, except that the models did not include stamen type and there was no continuous duration response variable.

We then re-ran the above anther and stigma contact models but with the addition of video length, total movement number and both variables as covariates to see if the increases in contact during both feeding visits were mediated by increased visit duration and/or number of movements from feeding on one stamen to another. We also grouped long and short feeding visits into single feeding visits for these analyses to better enable comparisons with the below analyses testing for differences in between-stamen versus within-stamen movements and because long and short feeding visits did not significantly differ in any of the prior analyses.

To test whether the number of movements within and between stamen types affected anther and stigma contact, we used the binary, count and continuous response variables listed previously, except that we only used binary contact with short stamen anthers since long stamen anthers were contacted in 96% of visits, providing insufficient power to test our hypotheses. In addition, we used the total duration of contact summed across short and long stamens and the count of body sections contacted by anthers of either stamen type, since our questions for these models were about total contact. We included the predictors movement number, movement type (within or between stamens), pollinator, and all two- and three-way interactions (except for binary anther contact, in which pollinator interactions were not included due to insufficient power), plus video length as a covariate. To test whether the effects we found of between-stamen-type movements on anther contact and total movement number on stigma contact differed by year, we ran additional models with each contact variable as the response and year, movement number (between stamens for anther contact, total for stigma contact), the interaction between year and movement number, pollinator and video length as the predictors.

To test for differences in dye particle deposition on bee bodies from short versus long stamen anthers, we used a normal linear regression with number of particles as the response and stamen type, body section, the interaction between stamen type and body section, and bee ID as predictors.

Table 1 summarizes our predictions under each hypothesis for the relationships between our predictor variables (stamen type, feeding type) and our measured response variables (pollen feeding, anther contact, stigma contact).

**Table 1.** Predicted results for the three hypotheses. A and B in hypothesis H1 are two alternative ways in which the hypothesis could be supported. Signs indicate the direction of effect of either stamen type (long stamens versus short stamens) or visit feeding type (long feeding versus short feeding versus both feeding) on the response variables of pollen feeding, anther contact and stigma contact. For example, ‘+long’ in the feeding column indicates the prediction of greater feeding on long stamen anthers, while ‘+short’ in the anther contact column indicates the prediction of greater contact between pollinators and short stamen anthers compared with long stamen anthers.

hypothesis		feeding	anther contact	stigma contact
H1. division of labour	A. long feeding	+long stamens	+short stamens	+long feeding
	B. short feeding	+short stamens	+long stamens	+short feeding
H2. pollinator specialization	taxon 1	+long stamens	+long stamens	+long feeding
	taxon 2	+short stamens	+short stamens	+short feeding
H3. movement		+both stamens	+both stamens	+both feeding

## 3. Results

### 3.1. Hypothesis H1: division of labour

**Figure 2.**
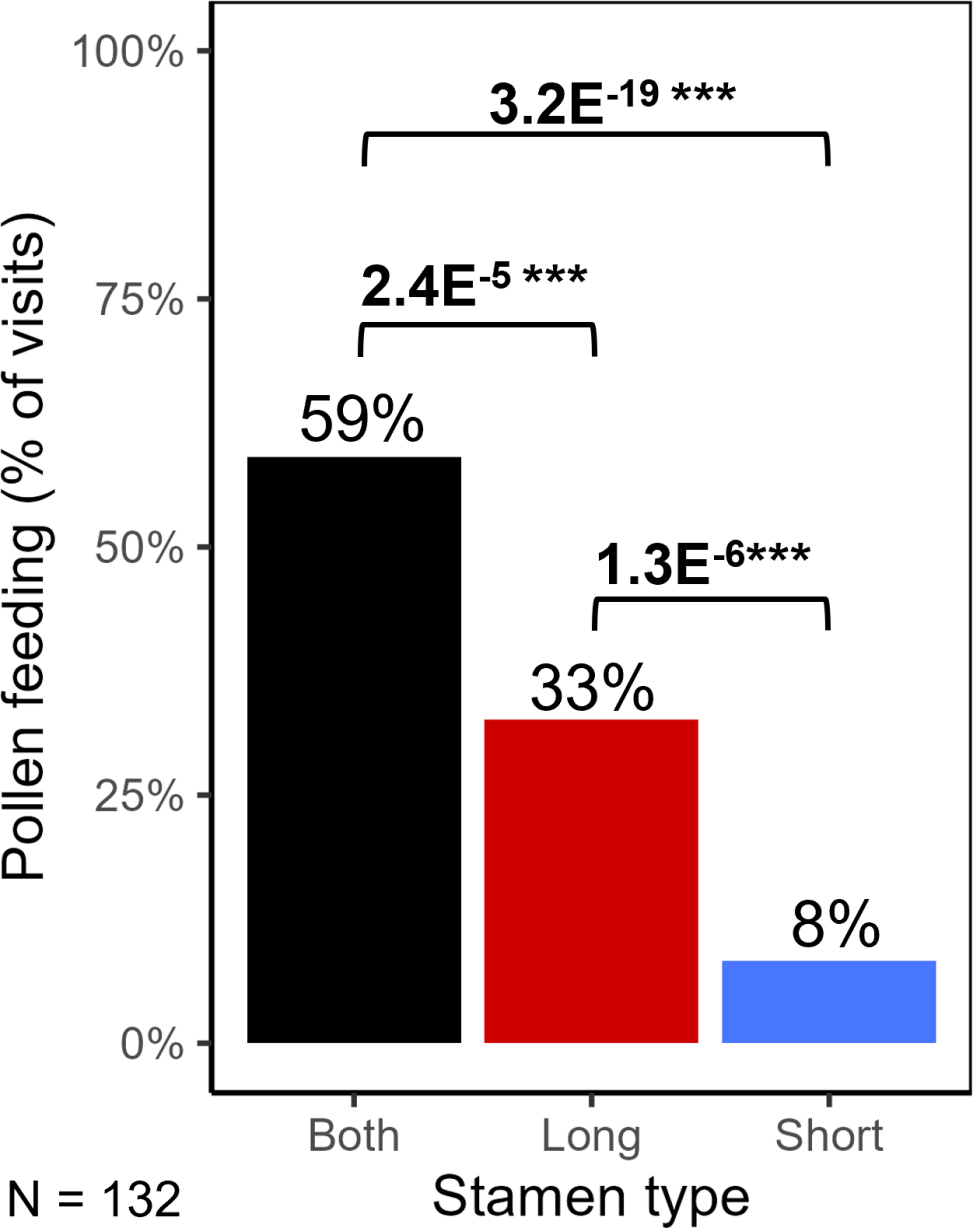
Proportion of the 132 recorded pollen-feeding visits that included feeding on both long- and short-stamen anthers, only long-stamen anthers or only short-stamen anthers. Numbers above brackets are *p*-values for the differences among bars from Fisher’s exact tests. Feeding frequencies did not significantly differ by pollinator taxon (χ^2^ = 1.77, *p* = 0.41).

From H1, we predicted that pollinators would feed mostly on one stamen type, positioning their bodies to better contact the anthers from the other stamen type (figure 1*b*). We found that pollinators were 2.9 times more likely to feed on only long-stamen anthers compared with only short-stamen anthers, but nearly 60% of pollen-foraging visits included feeding on both stamen types (figure 2), so anther types were not strongly specialized for feeding.

**Figure 3.**
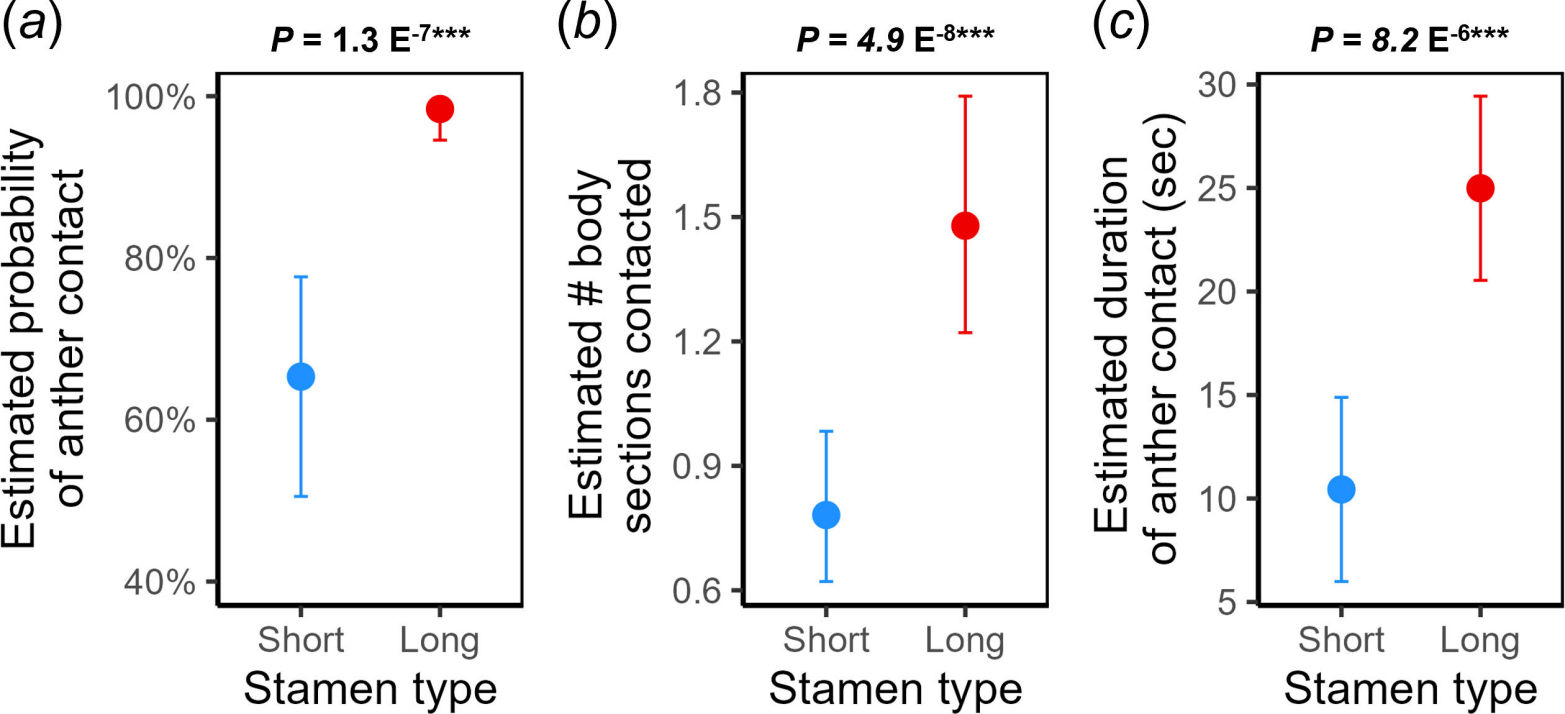
Effect of stamen type (short vs. long) on probability of anther contact (*a*), number of body sections contacted (*b*), and duration of anther contact (*c*) during 132 pollinator visits. Points are estimated marginal means after accounting for pollinator taxon and feeding type (see §2 and electronic supplementary material, tables S1 and S6 for model details), and error bars are 95% confidence intervals. *p*-values on top of each plot are for the stamen-type main effect from type III analyses of variance/deviance.

Despite this weak evidence for long stamens as feeding stamens, we tested the corresponding prediction that short stamens are specialized for pollinating (table 1, hypothesis H1A). Instead, we found that the long stamens were more likely to contact pollinator bodies overall (+33% of visits; figure 3*a*) and by body section (+0.7 sections contacted; figure 3). Long stamens also contacted pollinator bodies for longer durations overall (+15 s; figure 3*c*) and across body sections, but only significantly so for legs (electronic supplementary material, figure S1).

Aligning with our video analysis results, we found that there were an average of 4.1 times as many long stamen dye particles as short stamen dye particles deposited on small bees’ bodies in our preliminary gelatin swabbing test, a pattern that held across dorsal, ventral and side body sections (*n* = 3 bees and 2104 dye particles, electronic supplementary material, figure S2).

After failing to find evidence supporting the classic division of labour hypothesis involving feeding versus pollinating stamens, we tested two other variations on this idea. First, both stamen types may serve both functions but do so at opposite times as the other stamen type (i.e. longs pollinate during short feeding and vice versa). To test this, we examined patterns of contact during visits that included only feeding on short stamen anthers (*n* = 11, ‘short feeding’) versus only feeding on long stamen anthers (*n* = 43, ‘long feeding’). Contrary to our hypothesis, we found that pollinators were more likely to contact the stamen type they were feeding on, significantly so for long stamens (+75%, *p* < 0.001) but not short stamens (+18%, *p =* 0.31). Pollinators also contacted the stamen type being fed on for a longer duration in the case of long stamens (+25 s, *t*-ratio = 6.0, *p* < 0.001; compare red triangles to circles in electronic supplementary material, figure S3) or a similar duration in the case of short stamens (+0.8 s, *t*-ratio = 0.10, *p* = 0.87; compare blue circles to triangles in electronic supplementary material, figure S3).

**Figure 4.**
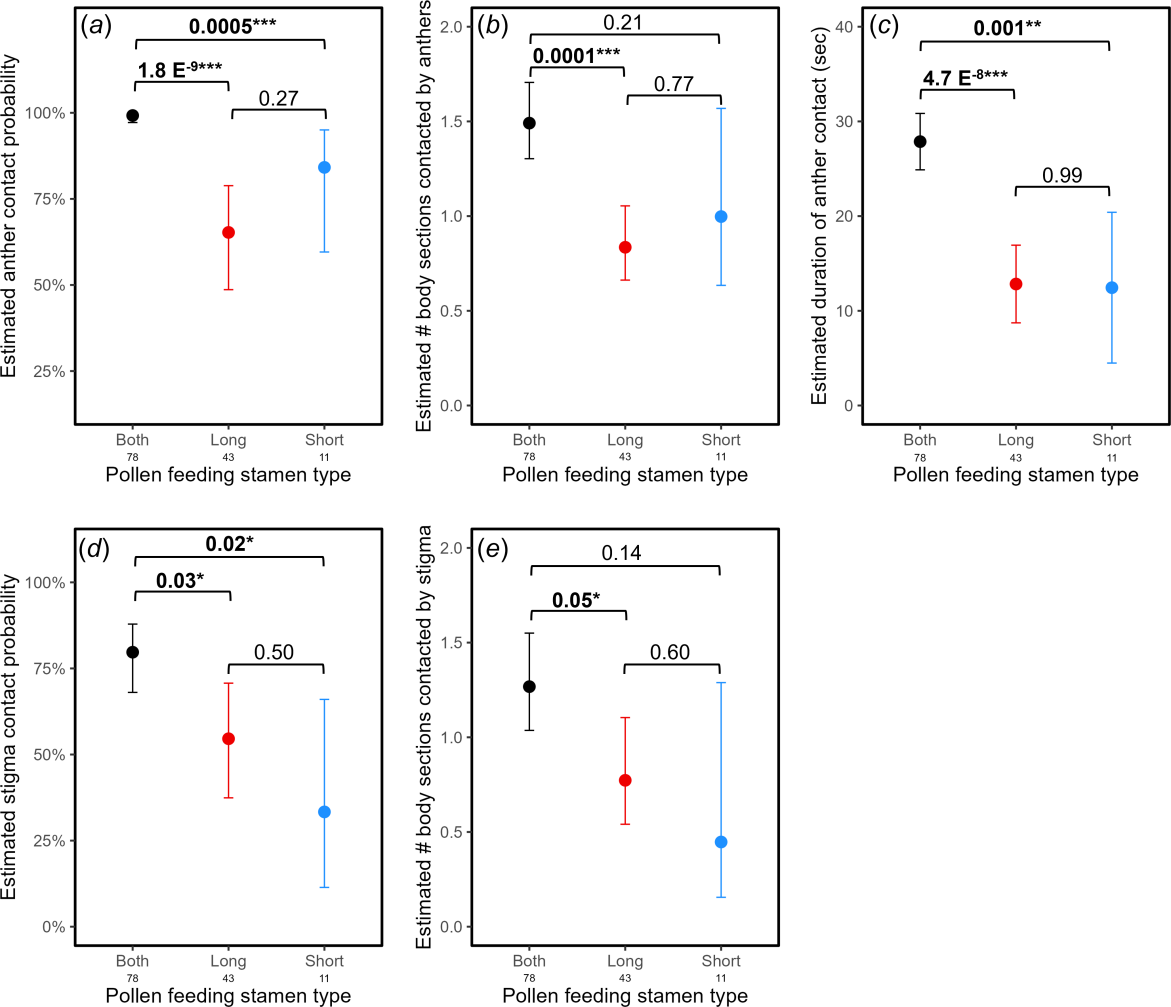
Contact with anthers and stigma by visit feeding type—both long and short stamen anther feeding or only one or the other. Probability (*a*), body section count (*b*) and duration (*c*) contact between anthers and pollinator bodies. Probability (*d*) and body section count (*e*) of contact between stigma and pollinator bodies (duration of stigma contact was not recorded; see §2). Points are estimated marginal means after accounting for pollinator taxon (small bee or syrphid fly) and contact stamen type (for *a*–*c*; see §2, electronic supplementary material, tables S1 and S6 for model details). In all panels, error bars are 95% confidence intervals. Numbers below *x*-axis labels are sample sizes. Numbers above brackets are *p*-values from Tukey post hoc comparisons for the three levels of feeding type.

Second, it is possible that feeding on one stamen type positions pollinators to better contact the stigma, while feeding on the other stamen type positions them to better contact the anthers, thus creating a division of labour between the pollen pickup and the deposition parts of pollination. However, long feeding and short feeding visits had similar levels of contact with both the anthers (figure 4*a-c*) and the stigma (figure 4*d,e*).

### 3.2. Hypothesis H2: pollinator specialization

From H2, we predicted that the two taxa included in our study, small bees and syrphid flies, would show contrasting patterns of feeding, anther contact and/or stigma contact by stamen type (figure 1*c*). However, we found no significant differences between pollinator taxa in terms of which stamens they fed on (χ^2^ = 1.77, *p* = 0.41). Across all visits, there were also no significant interactions between taxa and stamen type for the likelihood of contact (*p* = 0.68, electronic supplementary material, table S1), duration of contact (electronic supplementary material, figure S3) or number of body sections contacted (*p* = 0.71, electronic supplementary material, table S1). There was a weak but significant three-way interaction between pollinator taxa, pollen feeding stamen type and contact stamen type for anther contact duration (electronic supplementary material, figure S3). However, this interaction cannot support H2 in the absence of either of the two-way interactions with taxa. Finally, there was no significant effect of taxa in the differences in stigma contact by pollen feeding type for either proportion of visits (electronic supplementary material, figure S4) or number of body sections (*p =* 0.19, electronic supplementary material, table S1).

### 3.3. Hypothesis H3: movement

From H3, we predicted that pollinator movement from feeding on one stamen type to another would result in greater contact with the anthers and/or stigma (figure 1*d*). In line with our hypothesis, both feeding visits had greater anther contact than either long feeding or short feeding visits in terms of probability (figure 4*a*), body section count (figure 4*b*; both versus short not significant) and duration (figure 4*c*). Furthermore, pollinators were more likely to contact the stigma during both feeding visits overall (figure 4*d*) and marginally by body section (figure 4*e*).

**Figure 5.**
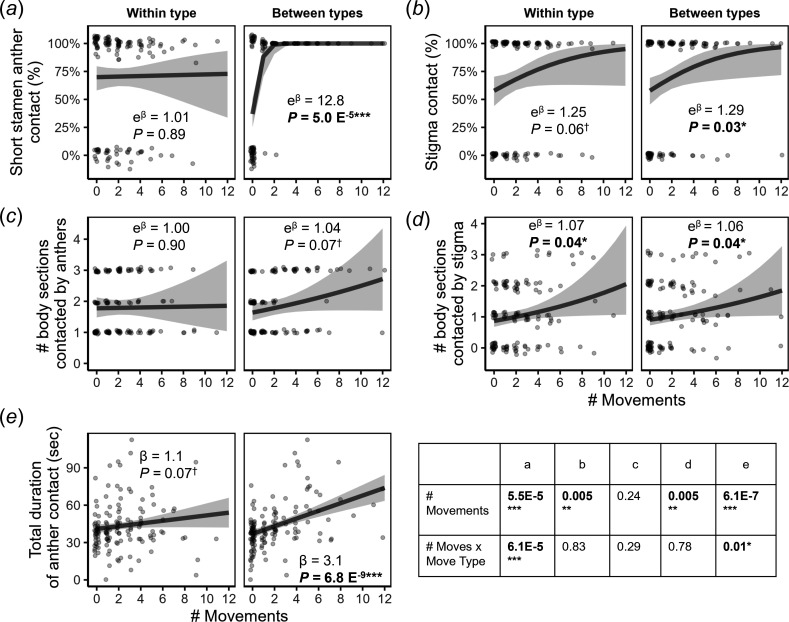
Effects of the number of movements from feeding on one stamen to another and the type of movement—within stamen type (short to short or long to long) or between stamen types (short to long or long to short)—on probability of short stamen contact (*a*), probability of stigma contact (*b*), number of body sections contacted by the anthers (*c*), number of body sections contacted by the stigma (*d*), and the total duration of anther contact (*e*). Dark grey lines are trend lines from the models, shaded areas are 95% confidence bands, and points are raw data adjusted for variation in visit length, jittered along the *x*-axis. The box shows the *p-*values for the effects of number of movements and the interaction between the number of movements and the movement type (testing for a difference in slopes) for the indicated panel. Models also included pollinator (small bee or syrphid fly), interactions with pollinator (except for a, see §2), and visit length. The beta coefficient and *p-*value for the effect of the number of movements are shown within each panel. For the non-continuous response variables (*a–d*), beta is exponentiated, giving the odds ratio (for the binary response variable in a and b) or rate ratio (for the count response variable in *c* and *d*). An odds ratio of 1 corresponds to a slope of 0.

In addition to categorizing visits by whether they included feeding on one or both stamen types, we also quantified the number of movements between stamen types that occurred during each visit. We predicted that the number of movements between stamen types would have a larger positive effect on anther and stigma contact than the number of movements within stamen types. This would be reflected in a larger positive slope for the effect of between-stamen movements compared to within-stamen movements, producing a significant interaction between number of movements and movement type. This prediction was confirmed for the probability (figure 5*a*) and duration of anther contact (figure 5*e*) and was in the predicted direction but not significant for the number of body sections contacted by anthers (figure 5). However, for stigmas, the probability of contact (figure 5*b*) and number of body sections contacted (figure 5) both increased on average with the number of movements regardless of the type of movement. On average, visits to the more pollen-depleted flowers in 2022 included a greater number of total movements even after accounting for pollinator taxon (+2.2 movements, *p* = 0.006), as expected by optimal foraging theory. However, the positive effects of between-stamen movements on anther contact (electronic supplementary material, table S4) and total movements on stigma contact (electronic supplementary material, table S5) held across years, with evidence for a significantly stronger effect in 2022 only for anther contact duration.

Considering both the categorical and quantitative results together, we tested whether the observed increases in anther and stigma contact during both feeding visits were mediated directly by the movement between stamen types or indirectly by an increase in visit length and/or total number of movements between stamens. To align with the within- versus between-movement analyses, and given the lack of significant differences between long and short feeding visits, we grouped long and short feeding visits into single feeding visits for these analyses. Both feeding visits were on average about 11 s longer than single feeding visits (*t* = 3.0, *p =* 0.004) and had 3.9 more total movements even after accounting for differences in visit length (*t* = 6.0, *p* < 0.001). Visit length and total movement number were moderately positively correlated (Pearson’s *r* = 0.35, *p* < 0.001). Both feeding visits still had significantly greater anther contact (for all three measures) after accounting for the effects of both visit length and total movement number (electronic supplementary material, table S2). This is consistent with our finding that between-stamen-type movements were an important predictor of anther contact (figure 4*a*,*c*,*e*). On the other hand, both feeding visits no longer had significantly greater stigma contact (for both measures) after accounting for total movement number (electronic supplementary material, table S3). This is consistent with our finding that the total number of movements among stamen types, but not the type of movement, was a significant predictor of stigma contact (figure 4*b*,*d*).

## 4. Discussion

### 4.1. No evidence for a division of labour between stamen types

Our first hypothesis was that there is a division of labour between short and long stamens in feeding and pollinating functions. If this hypothesis were supported, we expected to find that pollinators fed mostly on one stamen type, positioning their bodies to better contact the anthers from the other stamen type. Instead, we found that pollinators fed on both stamen types during over half of visits. While there was a slightly higher chance of feeding on long-stamen anthers, this anther type also had increased contact with pollinator bodies across all of our measures. These results are consistent with the findings of the prior studies, which show an increased proportion of pollen removed in single visits (through some combination of feeding and body contact) from long versus short stamen anthers in this species [17], tetradynamous *B. rapa* [20] and tristylous but non-heterantherous *Pontederia cordata* [36,37]. Our data also failed to support two other variations on the idea of dividing labour: stamen types serving both functions but at opposite times as the other stamen type or a division of labour between the pollen pickup and deposition parts of pollination. Thus, long stamens and short stamens seem to function similarly, with the four long stamens doing both more feeding and more pollinating than the two short stamens.

A family-level survey found strong associations between heteranthery and poricidal anthers (pollen is vibrated out of pores), bee pollination, lack of nectaries, enantiostyly (style deflected right or left), zygomorphy (bilateral symmetry) and relatively few stamens [8]. Wild radish flowers are partially bee-pollinated and have relatively few stamens but possess none of the other characteristics. In species with good evidence for a division of labour, feeding stamens tend to be shorter, centrally located, sterile and brightly coloured; pollinating stamens tend to be longer, fertile and cryptically coloured [9]. In contrast, both wild radish stamen types are fertile and yellow in colour. Although short stamens are less visible due to usually being tucked inside the corolla tube, our results show that this inconspicuousness does not accompany increased pollination function. Most of these characteristics of wild radish are largely consistent across other tetradynamous Brassicaceae species, likely explaining why they have generally not been considered heterantherous [8]. However, this study is the first to provide an experimental test of the hypothesis. Our results indicate that the intrafloral stamen differentiation occurring in most of the Brassicaceae family is different in kind from that occurring in heterantherous species in which feeding and pollinating functions are divided between stamen types.

### 4.2. No evidence that stamen types are specialized for different pollinator taxa

Wild radish flowers are primarily visited by the two pollinator taxa included in our study, small bees and syrphid flies. According to our pollinator specialization hypothesis, we predicted that these two taxa would show contrasting patterns of feeding, anther contact and/or stigma contact for short versus long stamens. However, we failed to find any evidence to support the idea that the two stamen types in wild radish are specialized to suit different taxa. These results are consistent with prior studies in wild radish finding no conflicting selection among pollinator taxa on four floral traits [23]. Further, the effects of anther separation and of the experimental removal of short stamens on single-visit pollen removal in wild radish did not differ across four taxa [17]. It remains possible that there is pollinator specialization on the two stamen types occurring for more granular taxonomic divisions, rarer taxonomic groups not included in this study (e.g. butterflies, wasps), or pollinator groupings based on morphology instead of lineage (e.g. body size, hairiness, proboscis length). Future studies might test these possibilities.

To our knowledge, interactions between pollinator types and tetradynamous stamen types have only been studied in wild radish. However, results from a few studies in species with other kinds of intrafloral stamen differentiation also largely fail to find support for the idea of pollinator specialization. In *Pterolepis glomerata*, both of the tested bee species removed more pollen from feeding than pollinating stamens [38]. In *P. cordata*, three bee species significantly differed in the proportion of pollen removed from long versus short stamen anthers in just one of three heterostylous morphs, and there was still no evidence for contrasting specialization in that morph [36]. In *Senna reniformis*, large bees preferentially fed on short stamen anthers, while small bees fed on anthers from all three stamen types indiscriminately [39]. Only one study suggests possible pollinator specialization for different stamen types, finding that Melastomataceae species with dimorphic compared with isomorphic stamen heights supported a greater diversity of pollinators; however, they could not separate the effect of stamen dimorphism from the correlated increase in flower size [40]. Therefore, the evidence so far suggests that multiple stamen types within flowers have not evolved to allow for more effective pollination by different taxa. A phylogenetic comparative study testing correlations between degree of pollinator specialization and presence or absence of tetradynamy in Brassicaceae would help test this hypothesis.

Although not the focus of this study, our results also suggest that small bees may be more effective pollinators of wild radish than syrphid flies, consistent with prior studies of pollen removal and deposition [26] and selection on floral traits [23]. Small bee bodies were slightly more likely to contact both short and long stamens and considerably more likely to contact the stigma compared with syrphid fly bodies.

### 4.3. Pollinator movement from feeding on one stamen type to another best explains the data

In this study, we propose a new hypothesis to explain tetradynamy: when pollinators move from feeding on one stamen type to another, the increased movement results in greater contact with the anthers and/or stigma, thus resulting in greater pollination effectiveness. Our results were consistent with this hypothesis, showing that visits in which pollinators fed on both stamen types had greater anther and stigma contact compared with those in which pollinators fed on only one stamen type.

This increased contact with anthers can primarily be explained by the direct effect of an increase in the number of movements between stamen types, pointing to a functional advantage of tetradynamy over uniform stamens. Movements between stamen types rather than within stamen types likely result in greater anther contact due to greater changes in pollinator position. While, to our knowledge, no prior studies have tested the consequences of feeding on multiple stamen types within a flower, a few studies have investigated the impact of pollinator body orientation on pollen pickup and/or deposition. For example, studies have found differences in pollen removal by bumblebee species that visited *Rhinanthus* [31] and *Agalinis* [41] flowers in an upright versus inverted orientation. In domesticated apple flowers, differences in seed set, pollen removal and pollen deposition have been found to vary based on whether bees forage for nectar from the top of the anthers or the petals [42,43].

On the other hand, increased contact with the stigma during visits that included feeding on both stamen types can be largely explained by the indirect effect of these visits including a greater number of total movements. It is possible that the presence of two stamen types tends to increase total pollinator movements, or that pollinators are more likely to feed on both stamens when moving around more. Therefore, while our results for stigma contact are consistent with the movement hypothesis for tetradynamy, they cannot rule out alternative hypotheses. While we are unaware of any prior studies that have looked for a relationship between the number of movements that occur during a visit and any aspects of pollination, a number of studies have looked at the length of time a pollinator spends visiting a flower. Although only moderately correlated in our data, the tendency for longer visits to involve more movement could explain why a positive relationship between visit length and pollen transfer has often been observed [22,44–46].

To gain further evidence for the movement hypothesis, future studies should pair slow-motion video analysis with single-visit counts of pollen swabbed from pollinators and deposited on stigmas. This approach would allow for testing the prediction that visits including feeding on both stamen types would have greater pollen pickup and deposition than visits including feeding on either stamen type alone.

## Data Availability

Data and code are publicly available from Dryad Digital Repository [47]. Supplementary material is available online [48].
